# Genipin and EDC crosslinking of extracellular matrix hydrogel derived from human umbilical cord for neural tissue repair

**DOI:** 10.1038/s41598-019-47059-x

**Published:** 2019-07-23

**Authors:** Karel Výborný, Jana Vallová, Zuzana Kočí, Kristýna Kekulová, Klára Jiráková, Pavla Jendelová, Jiří Hodan, Šárka Kubinová

**Affiliations:** 1 Institute of Experimental Medicine of the Czech Academy of Sciences, Prague, Czech Republic; 2 2nd Medical Faculty, Charles University, Prague, Czech Republic; 3 Institute of Macromolecular Chemistry of the Czech Academy of Sciences, Prague, Czech Republic

**Keywords:** Regeneration and repair in the nervous system, Mesenchymal stem cells

## Abstract

Extracellular matrix (ECM) hydrogels, produced by tissue decellularization are natural injectable materials suitable for neural tissue repair. However, the rapid biodegradation of these materials may disrupt neural tissue reconstruction *in vivo*. The aim of this study was to improve the stability of the previously described ECM hydrogel derived from human umbilical cord using genipin and N-(3-Dimethylaminopropyl)-N′-ethylcarbodiimide hydrochloride (EDC), crosslinking at concentration of 0.5–10 mM. The hydrogels, crosslinked by genipin (ECM/G) or EDC (ECM/D), were evaluated *in vitro* in terms of their mechanical properties, degradation stability and biocompatibility. ECM/G, unlike ECM/D, crosslinked hydrogels revealed improved rheological properties when compared to uncrosslinked ECM. Both ECM/G and ECM/D slowed down the gelation time and increased the resistance against *in vitro* enzymatic degradation, while genipin crosslinking was more effective than EDC. Crosslinkers concentration of 1 mM enhanced the *in vitro* bio-stability of both ECM/G and ECM/D without affecting mesenchymal stem cell proliferation, axonal sprouting or neural stem cell growth and differentiation. Moreover, when injected into cortical photochemical lesion, genipin allowed *in situ* gelation and improved the retention of ECM for up to 2 weeks without any adverse tissue response or enhanced inflammatory reaction. In summary, we demonstrated that genipin, rather than EDC, improved the bio-stability of injectable ECM hydrogel in biocompatible concentration, and that ECM/G has potential as a scaffold for neural tissue application.

## Introduction

Restoration of the neural tissue architecture and functions plays a crucial role in the treatment of the injured central nervous system (CNS). Several strategies have been developed and implemented in animal models to reconstruct the damaged neural tissue. One of these strategies – the tissue engineering approach - is bridging the lesion with the use of a biomimetic scaffold which is able to fill the lesion cavity and provide the necessary microenvironment for the neural tissue remodelling. Various synthetic and natural hydrogels have been used as tissue-engineered scaffolds for neural tissue repair^1–4^, but these materials usually fail to mimic the complex structure and composition of the native tissue. In contrast, biological scaffolds, composed of natural extracellular matrix (ECM), have many advantages including a three-dimensional (3D) structure, low immunogenicity and a complex biomolecular composition^5,6^.

Such scaffolds are commonly produced by whole tissue decellularization and may be prepared in injectable form by solubilisation of the obtained ECM into the hydrogel, using pepsin digestion at pH ∼ 2. These ECM hydrogels have physical properties (e.g. the ability to be physically crosslinked *in situ* at physiological pH and temperature) and adequate mechanical strength similar to the soft nervous tissue that allow them to be non-invasively injected into the defect caused by the injury with minimal surrounding tissue damage. ECM-based scaffolds, obtained from a variety of tissues, have been used for the reconstruction of e.g. myocardium, muscles, blood vessels, valves, bones, kidney, liver, as well as CNS tissues^7–9^.

In our recent study, we optimized the effective, reproducible decellularization techniques for the preparation of ECM-based hydrogel from human umbilical cord tissue and proved its neuro-promoting capacity, which was comparable to ECMs, derived from porcine tissues such as urinary bladder, spinal cord and brain^9,10^. In this context, due to neonatal and human origin, high accessibility and no ethical constraints, umbilical cord-derived ECM represents a promising tissue source for hydrogel preparation.

The control of ECM scaffold degradation is essential for the constructive tissue remodelling process, since the degrading biomaterial is gradually replaced by endogenous cells, which build a new functional ECM equivalent as opposed to scar tissue^5^. As shown in our previous studies and those of others, in inflammatory conditions the implanted ECM hydrogels are quickly populated with the resident cells, significantly accelerating the degradation rate of ECM-based scaffolds^10^. Therefore, the implementation of non-toxic, biocompatible and reproducible techniques to prolong and control the degradability of ECM-derived hydrogels, represents a challenging issue in applying such biomaterials for neural regeneration.

On the other hand, a long-term retention (12 weeks) but sparse endogenous cell invasion was found for ECM hydrogel derived from porcine urinary bladder injected into subacute stroke cavity in the brain, which indicates a marked difference of biodegradation of ECM hydrogel in the various types of CNS lesion^11^.

A common method to reduce the degradation of various biomaterials is chemical crosslinking. Formaldehyde or glutaraldehyde are common agents to stabilize ECM scaffolds^12,13^, however, the high cytotoxicity and potential induction of inflammatory response impedes these compounds the use as implants in neural tissue^14,15^. For that reason, non-cytotoxic crosslinkers, such as genipin or N-(3-Dimethylaminopropyl)-N′-ethylcarbodiimide hydrochloride (EDC) have been selected as candidates to improve the stability of ECM hydrogels^16,17^.

Genipin is a natural crosslinking low-toxic agent, derived from the gardenia fruit which can bridge free amino groups of lysine or hydroxylysine residues of different polypeptide chains by monomeric or oligomeric crosslinks in collagen^16–19^. To stabilize ECM scaffolds, genipin crosslinking was used in myocardial matrix hydrogel^20^, decellularized spinal cord^21^ or whole-liver decellularized grafts^22^, where it proved to have a lower cytotoxicity and *in vivo* immunogenicity than their glutaraldehyde-treated counterparts. In addition, genipin possesses a range of key pharmacological properties, such as anti-inflammatory, neuroprotective, neurogenic, and antidepressant effects which gives this compound therapeutic potential for diseases of the CNS^23^.

In contrast to genipin, EDC activates carboxyl groups of glutamic or aspartic acid residues to conjugate to amino groups between proteins and/or peptide molecules and is used for the crosslinking of proteins and polysaccharides. Collagen scaffolds crosslinked with EDC have been shown to decrease degradation rates^24,25^ while supporting the growth of human keratinocytes^26^, smooth muscle cells^27^, and fibroblasts^28^.

Therefore, due to the different reactions, fixation in genipin and EDC may produce distinct crosslinking structures that may affect the crosslinking characteristics, mechanical properties, and resistance against enzymatic degradation of the fixed material^16,17^.

In this study we used ECM derived from human umbilical cord, crosslinked by genipin and EDC to improve the structural stability, and evaluated feasibility of the obtained materials for neural tissue repair. Rheology, turbidimetry, and *in vitro* enzymatic degradation assays were used to characterize the kinetics of genipin and EDC cross-linking, and the effects of cross-linking on the gelation time, storage modulus and enzymatic degradation resistance. The *in vitro* biocompatibility of the crosslinked hydrogels was determined by the evaluation of human mesenchymal stem cell (MSCs) proliferation, axonal growth of dissociated adult rat dorsal root ganglion neurons and growth and differentiation of human fetal neural stem cells. In addition, *in situ* gelation and a prolonged retention of ECM/G hydrogels were confirmed after injection into a subacute photothrombotic cortical ischemic lesion in rats.

## Results

### Composition of ECM hydrogels

In our previous study we characterized the structure and composition of umbilical cord derived ECM hydrogel, while the amount of total collagen (543 μg/mg of dry ECM weight) and glycosaminoglycans (6.6 μg/mg of dry ECM weight) was determined^9^. In addition to this, the detailed proteomics of ECM matrix was performed in this study using high-resolution LC–tandem mass spectrometry and compared to the protein profile of the native umbilical cord tissue (Supplementary Table 1).

The composition of the ECM hydrogel confirms the previous conclusions on chemical compounds of ECM^9,29^. The most abundant proteins detected in the ECM are Collagen alpha-1(I) chain, Collagen alpha-2(I) chain, Collagen alpha-1(III) chain and Collagen alpha-1(II) chain, which were relatively enhanced in ECM in comparison with the native tissue. On the other hand, the decellularization process leads to a decrease of other types of collagen, such as Collagen alpha-1(IV, V, VI, XI, XII) chain, Collagen alpha-2(IV, V, VI, XI) chain or Collagen alpha-3(VI) chain, as well as to a substantial decrease of Fibronectin, Fibrillin-1, 2, Tenascin and Laminin subunit beta-1 and gamma-1. Some of the cytoskeletal or cytoplasmic proteins, such as Actin. aortic smooth muscle, Actin. cytoplasmic 2, Myosin-11, Filamin-A, Tropomyosin beta chain and alpha-1 chain, Caveolin-1, Myosin-10 and 9 were still preserved in ECM matrix, but to a very low extent (Supplementary Table 1).

### Crosslinking degree evaluation

**Figure 1:**
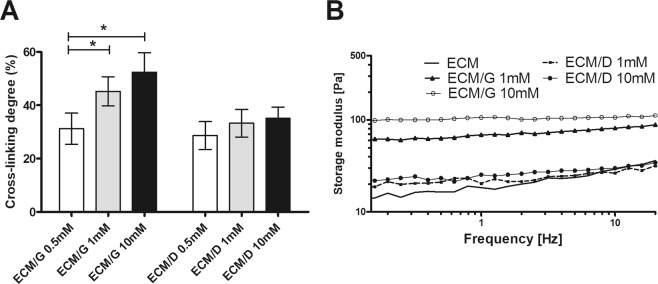
(**A**) The cross-linking degree and (**B**) strain sweep of ECM, ECM/G and ECM/D crosslinked by 0.5 – 10 mM genipin or EDC, (*p < 0.05, n = 3).

The 2,4,6-trinitrobenzene sulfonic acid (TNBSA) protocol was used to determine the relative amount of free amino group^30,31^. As is apparent in Fig. 1A, with the increasing concentration of genipin and EDC (0.5 mM; 1.0 mM and 10 mM), the crosslinking degree significantly increased from 31.3% to 52.5% in ECM/G but only from 28.6% to 35.1% in ECM/D. The uncrosslinked ECM keeps 0% baseline. The crosslinking degree of ECM/G increased with rising genipin concentration which shows that genipin reacts with the free amino groups in ECM and can form intra- and intermolecular crosslinking networks. On the other hand, EDC crosslinking efficiency is limited due to the need of both primary amine and carboxyl groups in the peptide or proteins. Moreover, EDC crosslinking is considered to be most efficient in acidic (pH 4.5) conditions while phosphate buffers and neutral pH (up to 7.2) conditions result in lower efficiency; nevertheless, an increasing amount of EDC in a reaction solution up to 10 mM was not able to compensate for the reduced crosslinking efficiency.

### Rheological and turbidimetric measurements

In rheological experiments, we tested the storage modulus to determine the flow and deformation properties of the crosslinked ECM hydrogels with crosslinkers concentration 1 mM and 10 mM. Increasing the frequency, the storage modulus of ECM and ECM/D retains nearly the same value and has an increasing trend. On the other hand, the storage modulus of ECM/G remarkably increased (Fig. 1B). These data reflect the lower efficiency of the ECM/D crosslinking degree assessed by the TNBSA protocol (Fig. 1A).

**Figure 2:**
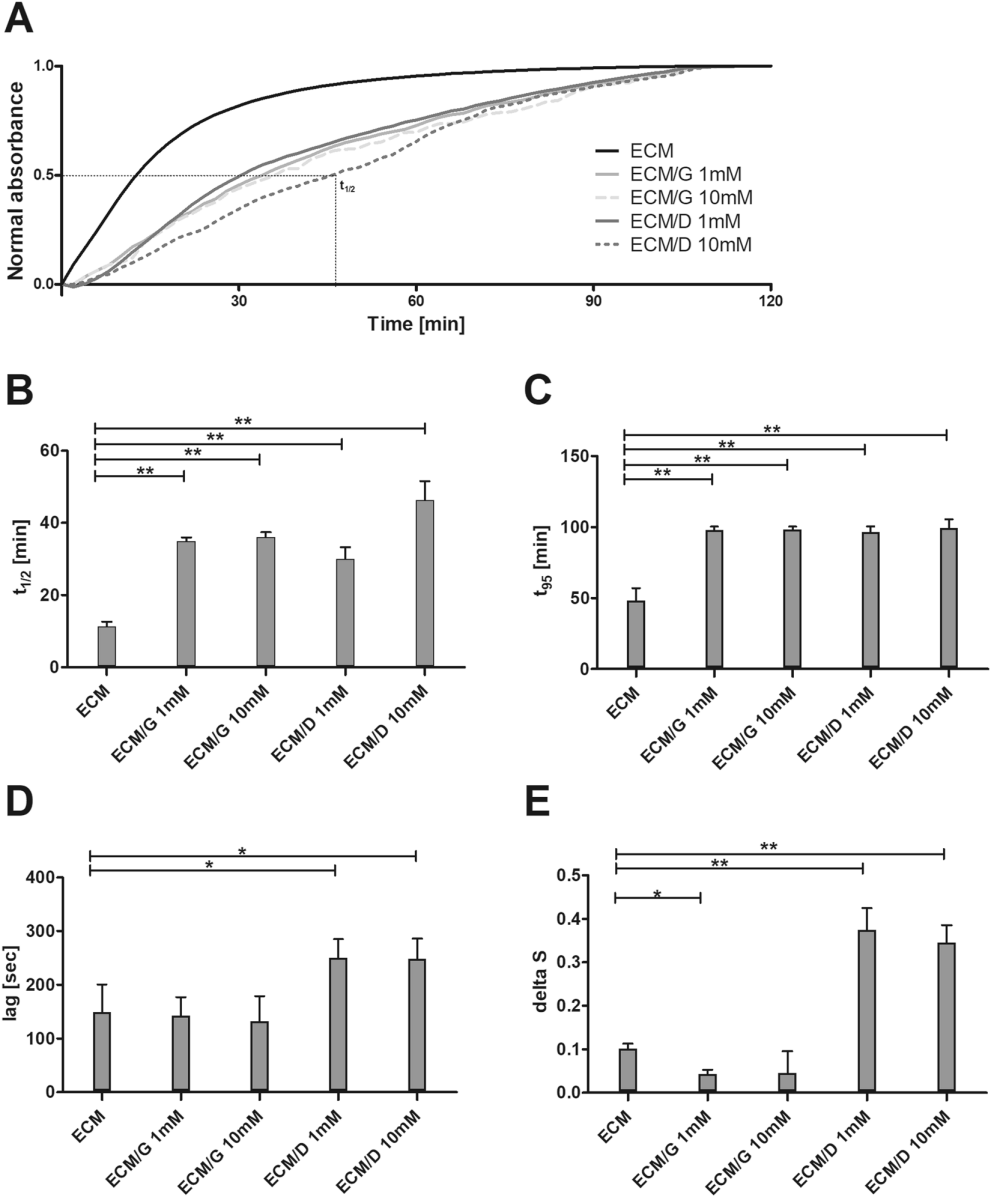
(**A**) The representative normalized turbidimetric gelation kinetics of ECM, ECM/G and ECM/D crosslinked by 1 mM and 10 mM genipin or EDC. (**B**) Time to reach 50% and (**C**) 95% maximal absorbance. (**D**) Lag time of ECM hydrogels determined as an intercept point of the slope at log t_1/2_ and turbidimetry baseline with 0% absorbance. (**E**) The gelation rate delta S defined as the slope of the linear region of the gelation curve (*p < 0.05, **p < 0.01, n = 3).

Turbidimetric gelation kinetic curves revealed a sigmoidal shape for uncrosslinked ECM and near-linear shape for ECM/G and ECM/D at a concentration of 1 mM and 10 mM (Fig. 2A). Notably, the gelation of uncrosslinked gel starts more rapidly than that for both the crosslinked ECMs and reached the plateau after ~100 min, compared to ~120 min for the crosslinked ECMs.

The time required to reach half of the final turbidity (t_1/2_) as well as the time required to reach 95% of the final turbidity (t_95_) was significantly longer for both crosslinkers at both tested concentrations than for the uncrosslinked ECM (Fig. 2B,C, Supplementary Table 2). On the other hand, a significantly higher lag time and gelation velocity at t_1/2_ (delta S) was found for ECM/D when compared with uncrosslinked ECM (Fig. 2D,E). These results suggest that hydrogel assembly was fastest for the uncrosslinked ECM while the crosslinking hydrogels required a longer gelation time.

### *In vitro* enzymatic degradation

**Figure 3:**
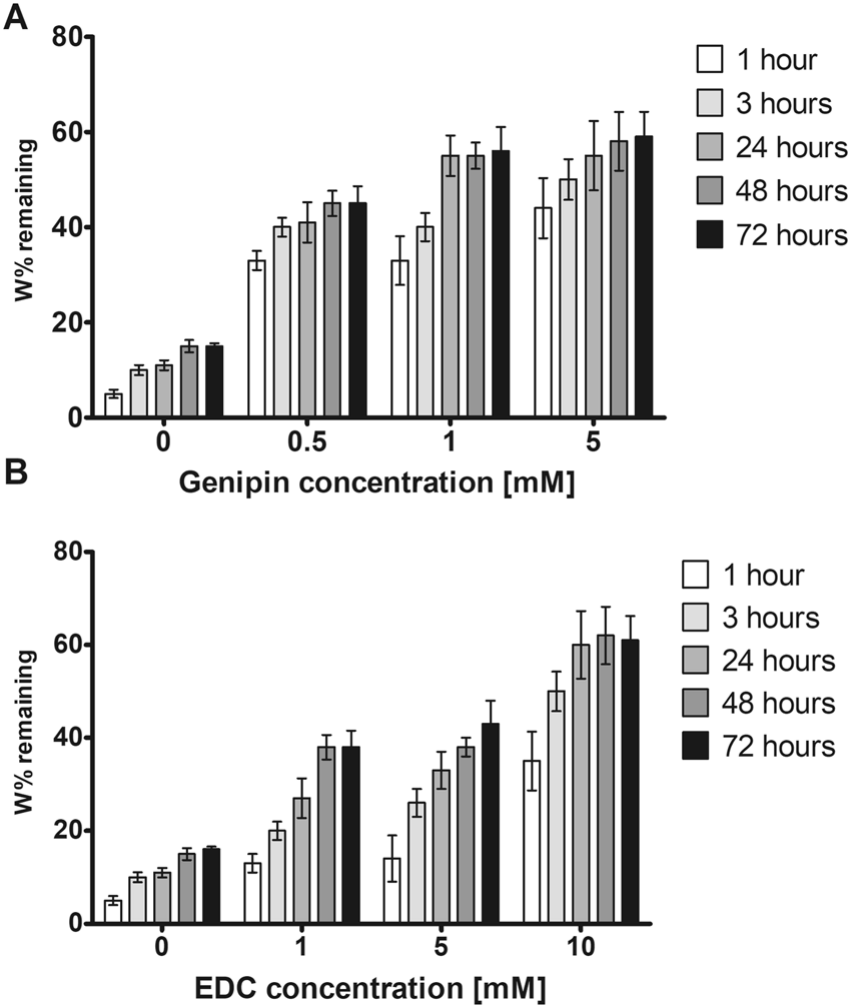
1-D enzymatic degradation of (**A**) ECM/G and (**B**) ECM/D in different concentrations of crosslinkers after hydrogel maturation time 1–72 hours. Data are reported as % of remaining weight expressed as the dry weight of the ECM hydrogels exposed to the collagenase for 3 hours related to the dry weight of control ECM gel exposed to PBS for the same time (n = 3).

1-D degradation test was used to analyse the ECM hydrogel endurance to enzymatic degradation in relation to the hydrogel maturation (Fig. 3). The % of the remaining weight was determined after 3 hours of exposure to 0.1% collagenase, while ECM, ECM/G and ECM/D hydrogels were allowed to mature for 1–72 hours. The collagenase concentration (0.1 wt %, 163 U/mg) was chosen to provide a standardized, accelerated assay for gel degradation in order to compare the effect of crosslinkers concentration on *in vitro* gel stability. It should be noted that the concentration of collagenase is much higher than of that which could be found *in vivo*, while the number of other matrix degrading proteases are present in the inflamed body setting. Generally, all concentrations of genipin and EDC provided a substantial resistance to collagenase-associated degradation, which increased with crosslinker concentration and maturation time, but a higher resistance and shorter maturation time were found for genipin rather than for EDC. After 24 h maturation and crosslinker concentration 1 mM, the degradation resistance was 2-fold higher in ECM/D and almost 4-fold higher in ECM/G than in the uncrosslinked ECM. Degradation of the hydrogels did not further increase after 24 h of maturation for ECM/G or after 48 h for ECM/D, which reflects that crosslinked ECM/G mature earlier than ECM/D, even in higher concentrations of the EDC.

**Figure 4:**
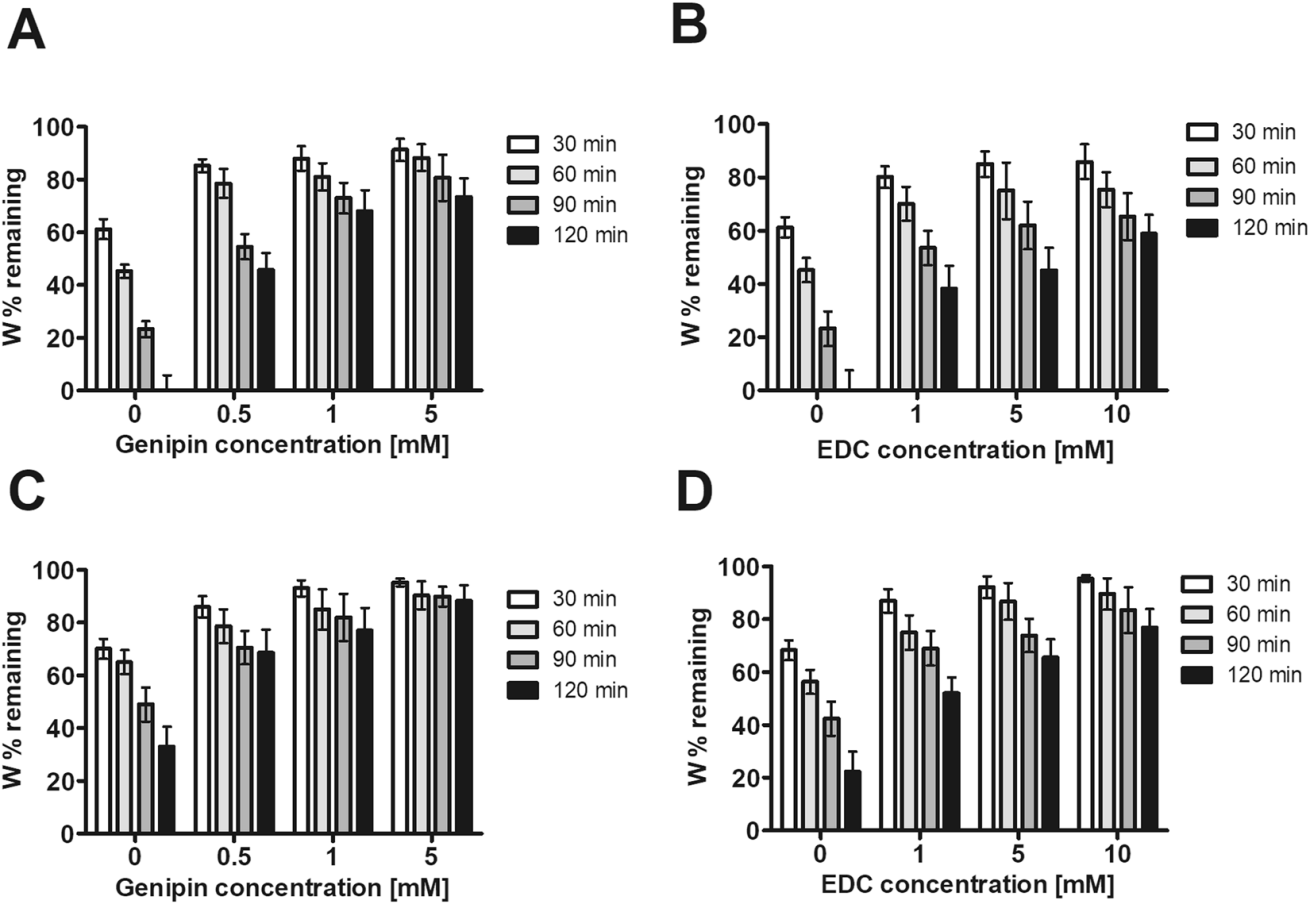
3-D degradation assay of ECM/G and ECM/D hydrogels in different concentrations of (**A**,**C**) genipin and (**B**,**D**) EDC, with a maturation time of (**A**,**B**) 1 hour and (**C**,**D**) 48 hours. Data are reported as % of remaining weight expressed as the dry weight of the ECM hydrogels after 30, 60, 90, 120 min exposure in collagenase solution related to the dry weight of control ECM gel exposed to PBS for the same time (n = 3).

A 3-D degradation model was used to further investigate the hydrogel’s stability (Fig. 4). ECM, ECM/G and ECM/D hydrogels were matured for 1 h (Fig. 4A,B) and 48 h (Fig. 4C,D) after the initial crosslinking and the degradation was determined as % of the initial weight after 30, 60, 90 and 120 min incubation in collagenase. ECM hydrogels which matured for 48 h showed a greater resistance to degradation than those which matured for 1 h, even in the case of uncrosslinked ECM. Similarly, as for the 1-D degradation, the resistance was higher for ECM/G than for ECM/D. Half-life calculations showed that ECM hydrogels matured for 1 h exhibited half-life times 96.37 min for 1.0 mM ECM/D and 170.38 min for 1.0 mM ECM/G hydrogels (Table 1).

**Table 1:** Half-life calculation of ECM, ECM/G and ECM/D hydrogels after 3-D degradation in 0.1% type I collagenase, (est.) estimated calculation using linear progression method, (n = 3).

Crosslinker concentration [mM]	Hydrogel type	Half-life time [min]	
		1-hour treatment	48-hours treatment
0	ECM	75.21 ± 8.27	43.52 ± 5.11
0.5	ECM/G	104.25 ± 6.86	234.11 (est.)
1	ECM/G	170.38 (est.)	310.82 (est.)
1	ECM/D	96.37 ± 8.54	130.11 (est.)
5	ECM/G	225.42 (est.)	605.21 (est.)
5	ECM/D	112.63 ± 6.41	173.89 (est.)
10	ECM/D	150.12 (est.)	305.82 (est.)

### Cell proliferation in the presence of genipin and EDC

**Figure 5:**
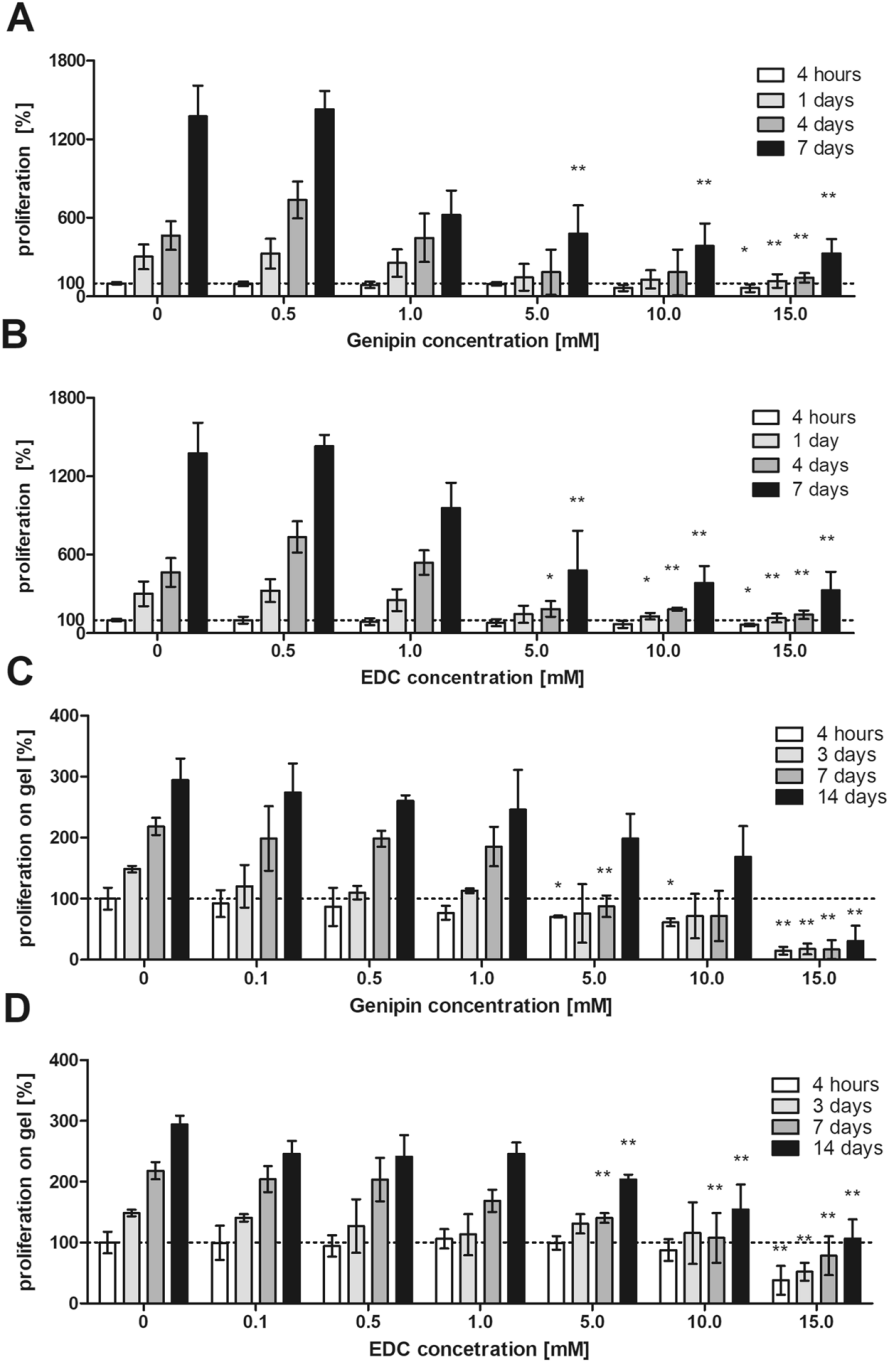
MSC proliferation in different concentrations of (**A**,**B**) free genipin and EDC, and (**C**,**D**) on ECM/G and ECM/D hydrogels crosslinked with different concentrations of genipin and EDC. Cell proliferation was assessed using Alamar Blue assay. The data are normalized to the initial values obtained at the beginning of the experiment on the uncrosslinked ECM which were set as 100% cell viability (dotted baseline), (*p < 0.05, **p < 0.01 vs. uncrosslinked ECM, n = 6).

Using MSC culture, various concentrations of free genipin and EDC added to the culture media were tested after 1, 4 and 7 days of the culture to evaluate the toxicity and effect of the crosslinkers on cell proliferation. As is apparent from Fig. 5A,B, proliferation of MSCs significantly decreased when the concentration of genipin as well as EDC in the culture media was 5 mM and higher.

### Cell proliferation on the crosslinked ECM hydrogels

The proliferation of MSCs seeded on ECM/D and ECM/G hydrogels with an increasing concentration of crosslinkers, was determined after 3, 7 and 14 days of the culture (Fig. 5C,D, Supplementary Fig. 1). A significantly decreased cell proliferation was found on the ECM hydrogels crosslinked with a genipin or EDC concentration of 5 mM and higher in comparison with the uncrosslinked ECM, while a crosslinker concentration of 1 mM was found biocompatible without remarkable effects on MSC proliferation. It is apparent that the cytotoxic effect of the crosslinked hydrogels occurs at a higher crosslinker concentration than in the case of free crosslinkers added into the culture media. The cytotoxic effect in higher crosslinker concentrations is most likely caused by the residual or unbound crosslinkers within the matrix.

### ECM hydrogel contraction

**Figure 6:**
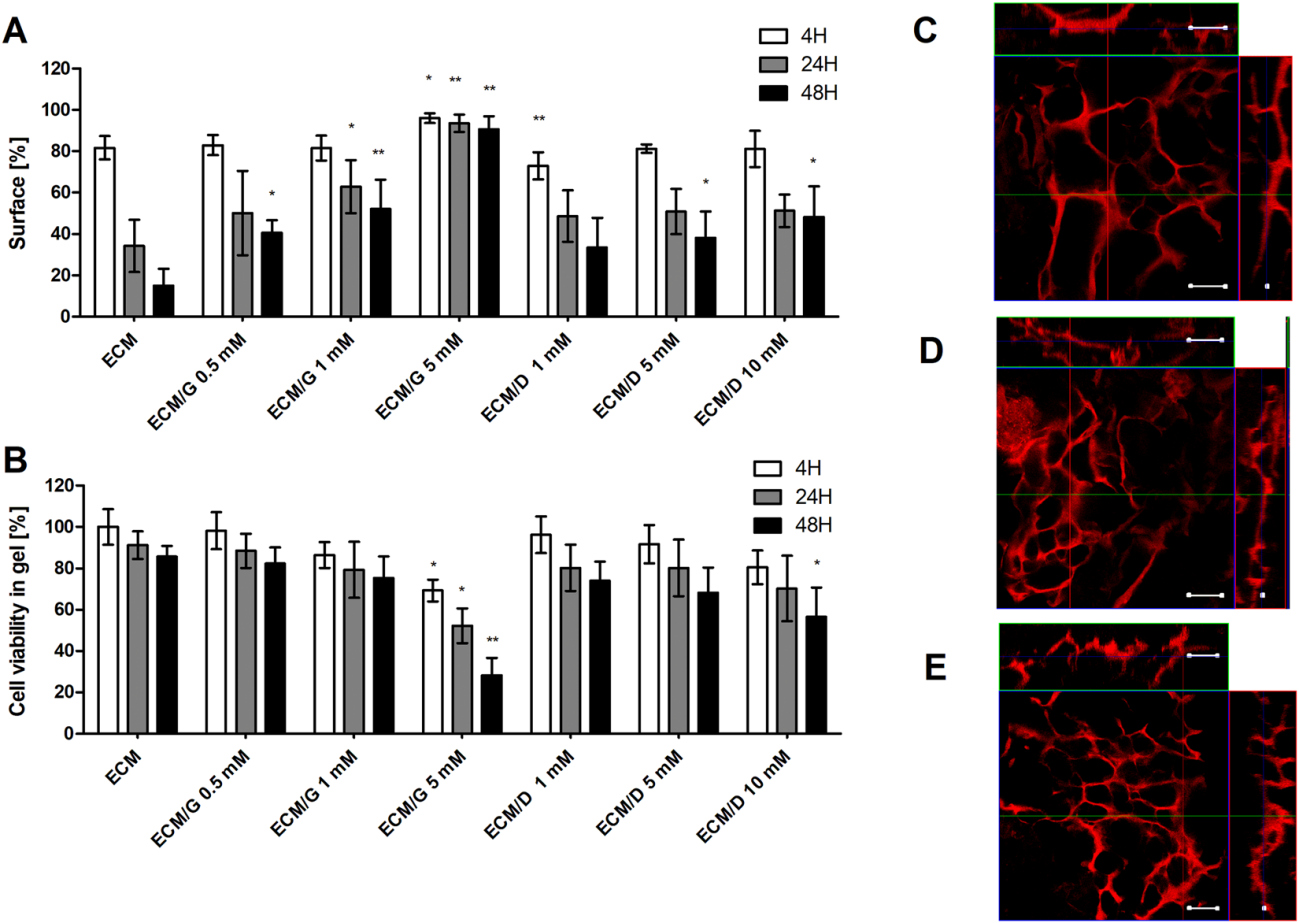
(**A**) Contraction of ECM, ECM/G and ECM/D hydrogel discs crosslinked with different concentrations of genipin and EDC, 4 h, 24 h and 48 h after 3D seeding with MSCs (5 × 10^5^ in 200 µl of the hydrogel). Contraction is expressed as a percentage of the initial hydrogel disc area (*p < 0.05, **p < 0.01 vs. uncrosslinked ECM, n = 3). (**B**) Viability of MSCs seeded in ECM, ECM/G and ECM/D hydrogels crosslinked with different concentrations of genipin and EDC at 4 h, 24 h and 48 h after 3-D cell seeding. Viability is expressed as a percentage of the initial cell viability in uncrosslinked ECM (*p < 0.05, **p < 0.01 vs. uncrosslinked ECM, n = 3). (**C**–**E**) 3-D confocal micrographs of MSCs stained for phalloidin seeded on (**C**) ECM, (*D*) 1 mM ECM/G and (*E*) 1 mM ECM/D after 7 days of the culture. Scale bar: 100 µm.

The mechanical stability of the crosslinked ECM hydrogels was evaluated in a 3-D cell culture, which result in the hydrogel contraction. Figure 6A depicts a contraction of ECM hydrogels seeded by MSCs (5 × 10^5^ in 200 µl of the hydrogel disc) after 4 h, 24 h and 48 h. In uncrosslinked ECM hydrogels, a significant contraction leads to the shrinkage of the hydrogel disc area to 24% within 24 hours. However, crosslinking of the ECM hydrogels by 1 mM genipin or EDC reduced the contraction to 49.9% (ECM/G) and 51.3% (ECM/D) (within 24 h) of the initial hydrogel disc area, while using this concentration, no decrease in cell viability within the ECM hydrogels was observed (Fig. 6B). The contraction was blocked with the genipin crosslinking of concentrations of 5 mM. On the other hand, EDC did not prevent hydrogel contraction, even at a concentration 10 mM of EDC. At concentration of genipin of 5 mM, and EDC of 10 mM, the viability of MSCs in 3-D cell culture significantly decreased (Fig. 6B). These findings are in line with the results from the mechanical strain which was improved after genipin but not after EDC crosslinking (Fig. 1B).

The ability of MSCs to grow from the surface into the ECM hydrogels was observed after 1 week of the culture (Fig. 6C–E). The cells formed an interconnected 3-D network within the uncrosslinked ECM as well as in ECM/G and ECM/D crosslinked by 1 mM genipin or EDC.

### The effect of crosslinking on axonal growth

**Figure 7:**
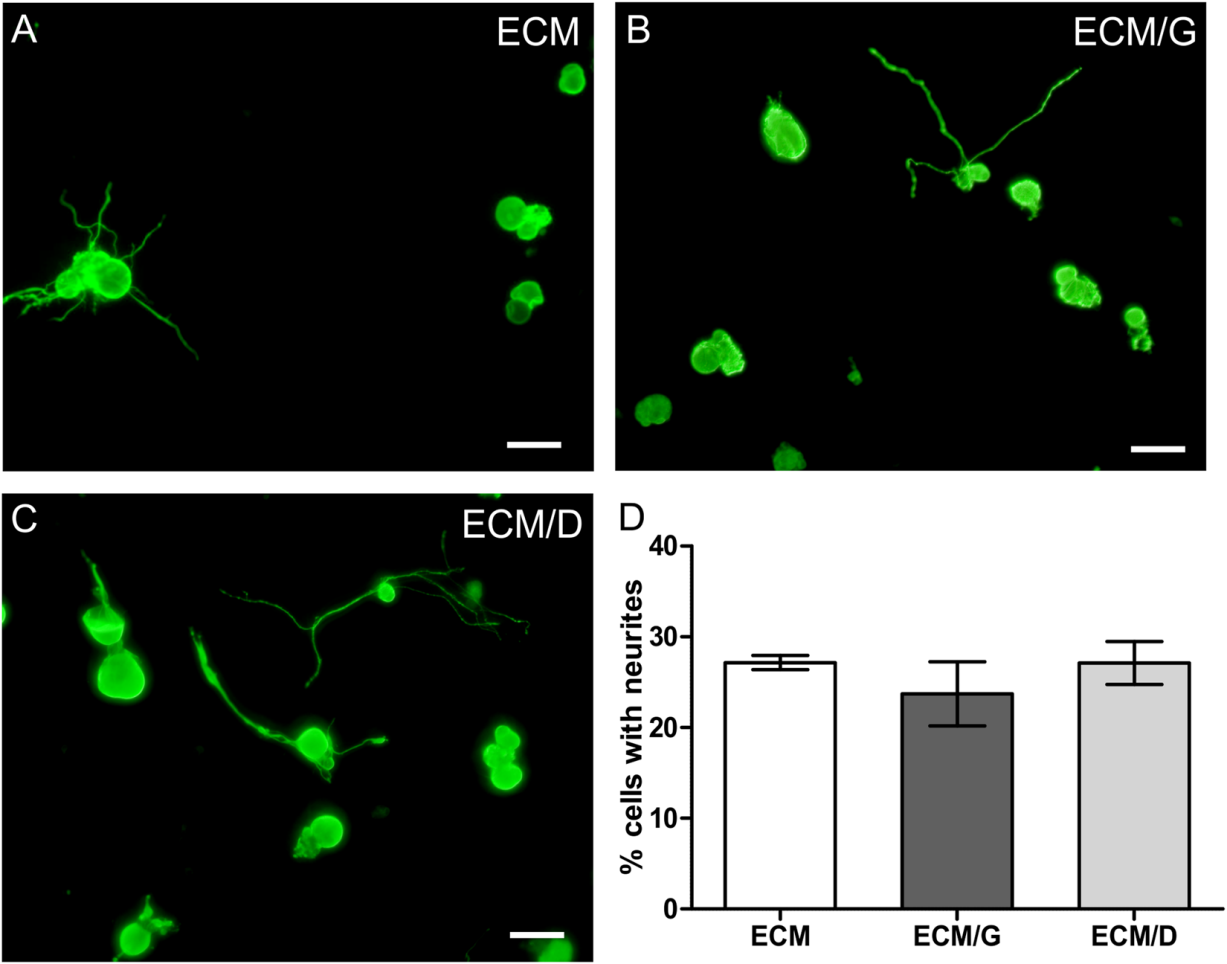
Axonal growth of dissociated adult dorsal root ganglion (DRG) neurons on the ECM, ECM/G and ECM/D hydrogels with crosslinker concentration 1 mM. (**A**–**C**) DRG culture on ECM hydrogels stained for beta-III-Tubulin. (**D**) Percentage of cells with neurites longer than cell body. Scale bars represent 50 µm (n = 3).

The effect of crosslinking of ECM hydrogel with 1 mM genipin and EDC was evaluated by analysis of the axonal growth of sensory neurons isolated from adult DRG. The percentage of neurons that displayed axonal growth was much lower than on the laminin coated glass cover slips (not shown) but did not show any significant differences among the uncrosslinked ECM, ECM/D and ECM/G (Fig. 7).

### The effect of crosslinking on neural stem cell growth and differentiation

**Figure 8:**
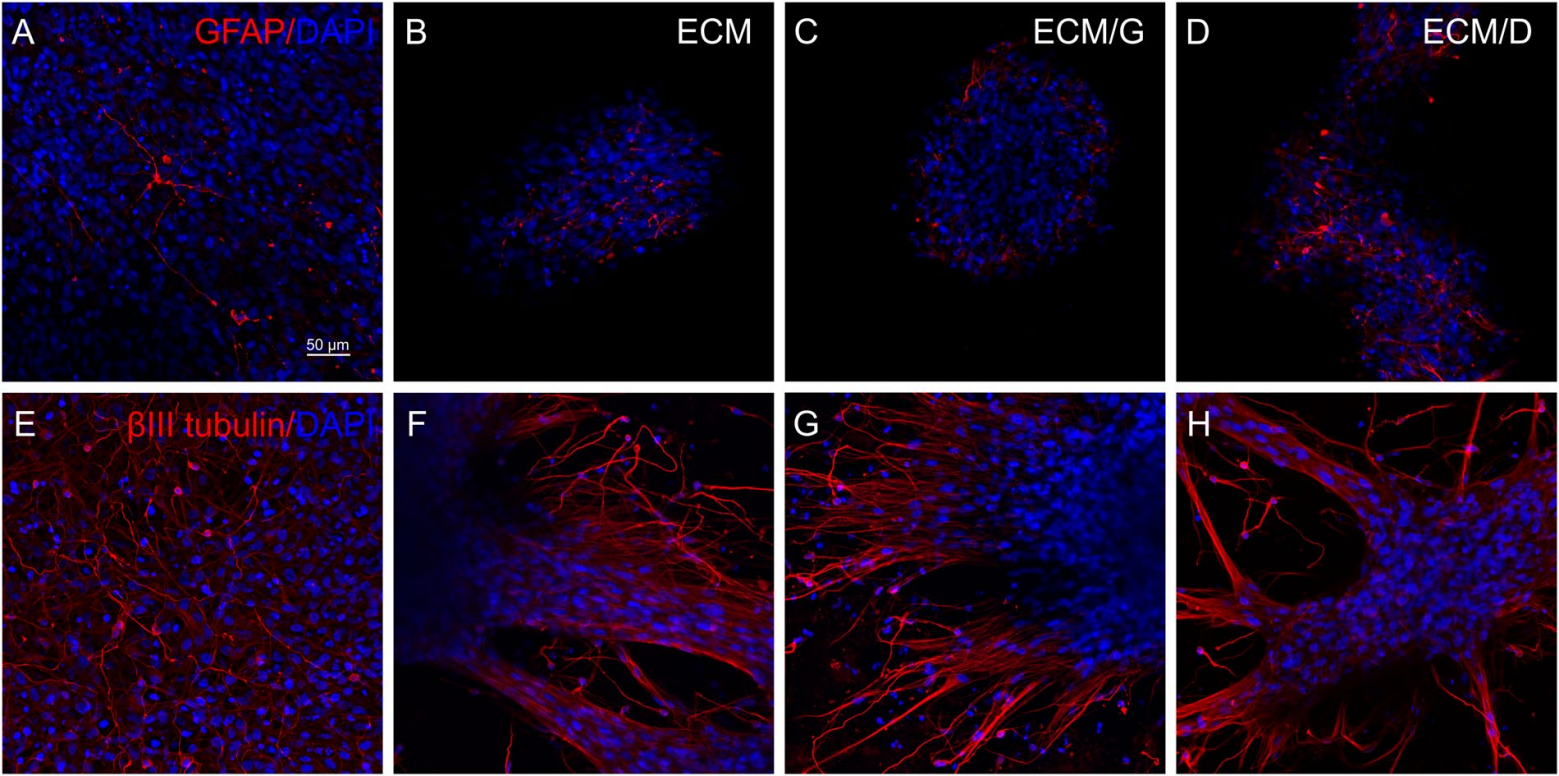
Neural stem cells (SPC-01) cultured (**A**,**E**), on the laminin coated glass coverslips, (**B**,**F**) uncrosslinked ECM, (**C**,**G**) ECM/G and (**D**,**H**) ECM/D. Cells were stained with (**A-D**) GFAP (red) and DAPI (blue) or (E-H) beta-III-Tubulin (red) and DAPI (blue). Scale bar: 50 µm.

The proliferation of neural stem cells (SPC-01 line) was evaluated after 1 and 3 weeks of the culture on uncrosslinked ECM, ECM/D (1 mM) and ECM/G (1 mM). After 1 week, SPC-01 grew on all ECM hydrogels in distinct clusters (Supplementary Fig. 2). The values of cell density on various ECM hydrogels did not significantly differ at both time intervals (Supplementary Fig. 3). The differentiation of SPC-01 was then analysed after 3 weeks in culture. SPC-01 cells spread from the clusters and covered large areas on hydrogels and were positive for neuronal marker beta-III-Tubulin, mainly on the borders of spreading clusters. The minority of cells were also positive for astrocytic marker GFAP (Fig. 8). However, the cells did not tend to differentiate into oligodendrocytes. There were only individual cells positive for NG2 at this interval, either on ECM hydrogels or laminin coated coverslips (not shown).

### *In vivo* evaluation of ECM/G hydrogel

**Figure 9:**
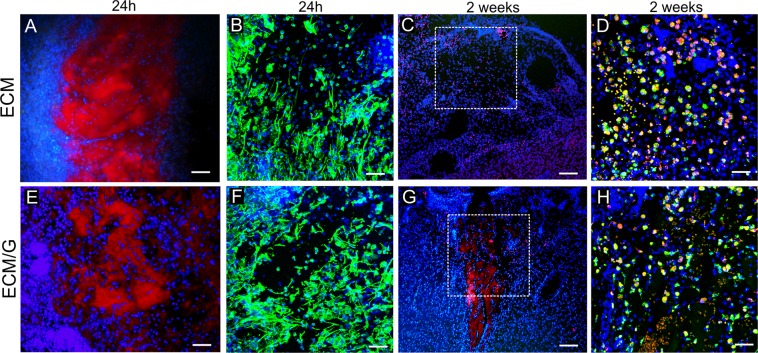
Coronal brain sections illustrating *in vivo* gelation and cellular infiltration of (**A**–**D**) ECM and (**E**–**H**) ECM/G hydrogel, (**A**,**B**,**E**,**F**) 1 day and (**C**,**D**,**G**,**H**) 2 weeks after the injection into the photothrombotic ischemic lesion in the rat motor cortex. (**A**,**C**,**E**,**G**) Staining for collagen I (red) and cell nuclei (DAPI, blue). (**B**,**F**) Staining for vimentin (green) and DAPI (blue). (**D**,**H**) The infiltration of macrophages (ED1, green) into the lesion with (**D**) uncrosslinked ECM hydrogel and (H) ECM/G 2 weeks after hydrogel injection into the lesion area. Cell nuclei were stained for DAPI (blue). The dotted squares in C, G show the area taken in D, H. Scale bars: (**A**,**E**,**C**,**G**) 100 µm, (**B**,**D**,**F**,**H**) 50 µm.

To prove the *in vivo* biocompatibility and degradation, ECM and ECM/G hydrogels were injected into the focal ischemic lesion created in the rat motor cortex to crosslink *in situ*. As is illustrated in Fig. 9A,E on collagen staining after 24 h, both ECM and ECM/G formed a compact hydrogel within the lesion and this was populated by the host cells, such as fibroblasts or macrophages. A remarkable infiltration of vimentin positive cells was observed in both the ECM and ECM/G hydrogels (Fig. 9B,F). The ECM and ECM/G hydrogel retention was then determined 2 weeks after the injection into the lesion. While uncrosslinked ECM degraded (Fig. 9C), ECM/G was still detected within the lesion without any sign of enhanced inflammatory reaction of fibrotic scarring (Fig. 9G,H). It is obvious that genipin crosslinking effectively enhanced ECM hydrogel retention within the lesion, while a gradual degradation was apparent in the surrounding tissue where the ECM/G fragments were highly infiltrated with the host cells. Unfortunately, due to the fragility of the lesion area, we were not able to perform a quantitative analysis that would reveal a hydrogel effect on cavitation and cell infiltration.

To determine the inflammatory reaction around the lesion treated by the ECM and ECM/G hydrogel, a relative number of microglia/macrophages positive for CD68 (ED1) and CD206 in the area surrounding the lesion were analysed after 2 weeks (Table 2). No significant differences were found between the group of ECM and ECM/G in the relative number of both ED1 and CD206 positive cells, which suggests that the genipin crosslinking of ECM hydrogel did not enhance an inflammatory reaction within the ischemic cortical lesion.

**Table 2:** The percentage of ED1 and CD206 positive cells in the lesion area after ECM and ECM/G injection into the ischemic cortical lesion. (n = number of animals).

	ECM	ECM/G
ED1 (% cell number)	33.84 ± 1.09 (n = 5)	35.56 ± 3.17 (n = 5)
CD206 (% cell number)	28.05 ± 2.97 (n = 5)	30.09 ± 1.91 (n = 5)

## Discussion

Decellularized ECM tissues can be transformed to scaffolds of natural origin in a variety of applications in tissue engineering^32^. To remodel neural or other soft tissues, ECM in the form of hydrogels are acceptable in clinical practice as these materials offer minimally invasive delivery techniques using the advantage of injectability and retention of biologic activity.

In our previous study, we prepared and characterized ECM hydrogel derived from the human umbilical cord and found it comparable to ECM hydrogels derived from CNS and non-CNS porcine tissues^9^. However, despite the advantageous bioactive properties, physically crosslinked ECM hydrogels exhibit weak structural stability and rapid *in vivo* degradation when applied into acute or subacute CNS lesion, which in turn limit their use for *in vivo* application^10^. On the other hand, a slowing degradation may prolong the presence of the bioactive ECM matrix within the CNS lesion and thus allow the resident cells and axons to repopulate the ECM scaffold, enabling tissue remodelling and functional bridging of the lesion.

To solve this problem, in this study we aimed to enhance the *in vivo* degradation resistance of the ECM hydrogels by covalent crosslinking, and evaluate the feasibility of the crosslinked materials *in vitro*, with the focus being on their biocompatibility and neuro-promoting potential.

We compared two crosslinkers, genipin and EDC, which due to their relatively low cytotoxicity are commonly used for the stabilization of natural scaffolds, e.g. collagen, gelatine, chitosan or ECM matrices^19,33^. Both crosslinking agents bind proteins or peptides, such as collagen which is the most prominent compound of ECM. Furthermore, the ECM scaffold is composed of a substantial amount of glycosaminoglycans as well as other proteins that may form an additional substrate for the crosslinking process^9^.

Importantly, the different principles of both crosslinking reactions and structures reflect the differences in the crosslinking degree and overall stability. While genipin primarily binds free amino groups with a wide variety of covalent cross-links, EDC requires carboxyl groups and binds primary amino groups through an active O-acylisourea intermediate.

A higher crosslinking degree which elicits genipin rather than EDC has a substantial impact on the enhancement of storage modulus and the resistance of ECM hydrogels to enzymatic degradation. Moreover, a lower degree of crosslinking of the EDC-fixed tissue in comparison with genipin was also described in previous reports^16,17,34^. Notably, N-hydroxysuccinimide (NHS) has been reported to improve the efficiency of crosslinking collagen in conjunction with EDC. However, due to the organic origin and potential toxicity of NHS when used in the *in-situ* ECM gelling process, we did not use EDC coupling with NHS or sulfo-NHS agents.

The effect of genipin crosslinking on the enhancement of ECM hydrogel mechanical strength was further confirmed by the weakening hydrogel contraction after seeding with MSCs in 3D culture. The contraction of ECM matrices after the inoculation of MSCs or other fibroblast-like cell types represents a common phenomenon for collagen-based materials that might burden the ability of the matrix to fill the lesion cavity^7^. Crosslinking with 1 mM genipin significantly decreased ECM contraction without affecting MSC viability and thus improved the hydrogel ability to effectively fill the lesion when combined with MSCs.

When developing injectable hydrogels, the crosslinker dosage is limited by its toxicity as the ECM hydrogels have to crosslink *in situ* without the possibility of washing out the excess of crosslinkers from the matrix. While the genipin is covalently bound within the crosslinked matrix, EDC serves as an intermediate substance which does not take part in the linkage and remains within the scaffold in the form of isourea byproduct. This might limit the EDC as a crosslinker *in vivo*, as in effect it requires a higher concentration for the efficient crosslinking than genipin. In this study, we used crosslinkers concentration 1 mM that was found to be noncytotoxic. This concentration provided a significant ECM hydrogel resistance to *in vitro* degradation without a deleterious effect on cell proliferation, axonal sprouting, and neural stem cell growth and differentiation. The support of crosslinked ECM hydrogels for neural stem cells and MSCs is of great importance as proof of the neuro-promoting properties of ECM hydrogels as well as for cell delivery, as these cell types have been shown to enhance the therapeutic benefit of the ECM scaffolds^10,35^.

To prove crosslinked ECM hydrogel stability *in vivo*, ECM and ECM/G were injected into the ischemic lesion to evaluate its retention within the lesion as well as the reaction of the host macrophages/microglia. Certainly, both ECM and ECM/G hydrogels formed a gel *in situ* and were infiltrated with the host cell, while genipin crosslinking enhanced ECM/G retention within the lesion for up to 2 weeks, when compared with uncrosslinked ECM hydrogels.

It has been reported that when applied *in vivo*, crosslinked collagen-based matrices induced a proinflammatory response, such as macrophage activation and the increase of proinflammatory cytokine release, while the host adverse response depends on the crosslinking degree^36^. In this study, the macrophage reaction did not reveal any significant changes that would indicate enhanced immunogenicity of ECM/G when compared to uncrosslinked ECM. Moreover, the prevalence of M2-like CD206 + macrophages was found in both ECM and ECM/G groups, similarly as in our previous reports^9,10^. This suggests that when exposed to the inflammatory environment of the subacute ischemic cortical lesion, the biocompatible concentration of genipin crosslinking (1 mM) was effective for *in situ* gelation and the prolongation of ECM hydrogel resistance without enhancement of inflammation reaction.

Apart from stabilization of the collagen matrix, genipin has been shown to have a significant effect on preventing cellular outgrowth^37^. In this study, we observed that MSCs were able to growth from the surface into the crosslinked ECMs *in vitro*. *In vivo*, the sparse host cell infiltration into preserved ECM/G was observed after 2 weeks. Nevertheless, gradual degradation of the ECM/G matrix and its replacement by endogenous tissue structures is apparent in the surrounding tissue. Therefore, longer time intervals are required to further assess the complete ECM/G degradation within the CNS lesion.

In summary, our study used genipin and EDC as crosslinkers to enhance ECM hydrogel stability. Both crosslinkers are well tolerated by various stem cells at concentrations of 1 mM, while genipin produced more stable hydrogels with a higher degree of crosslinking, enhanced storage modulus and resistance to *in vitro* degradation. Moreover, when crosslinked *in situ*, genipin prolonged the retention of the ECM/G hydrogel within the cortical ischemic lesion without any adverse or proinflammatory effects in the host tissue. To evaluate the feasibility of the ECM/G hydrogel in neural tissue repair, further *in vivo* study is required on the more appropriate models of CNS injury, such as SCI.

## Conclusion

Injectable ECM hydrogel derived from human umbilical cord was stabilized with genipin or EDC crosslinking. Genipin, rather than EDC, improved the mechanical strength and bio-stability of the ECM hydrogel, when compared to uncrosslinked ECM. A concentration of 1 mM of both genipin and EDC was found biocompatible without affecting the ability of ECM hydrogels to support axonal growth and neural differentiation. *In vivo*, 1 mM genipin was suitable for the *in situ* ECM crosslinking and improved retention of ECM/G in the cortical ischemic lesion for up to 2 weeks when compared to uncrosslinked ECM.

## Methods

### Tissue decellularization and preparation of ECM hydrogels

Human umbilical cords were prepared according to the previously described protocol^9^. The cords were obtained from healthy full-term neonates after spontaneous delivery with the informed consent of the donors using the guidelines approved by the Institutional Committee at University Hospitals (Pilsen, Czech Republic). About 10–15 cm of frozen umbilical cord was aseptically transported to the lab, subsequently thawed and transversely cut into pieces (<0.5 cm length). The tissue pieces were agitated in PBS bath (48 h at 120 rpm, 4 °C). The deionized water (dH_2_O) and PBS bath were exchanged three to five times before the tissue pieces were soaked in 0.02% trypsin/0.05% EDTA (120 min at 120 rpm, 37 °C, Sigma-Aldrich, Chemie GmbH Steinheim, Germany) and afterwards in a 0.1% peracetic acid in 4.0% ethanol bath (120 min at 300 rpm;) and in a series of PBS and deionized water (dH_2_O) soaks. Finally, the tissue pieces were lyophilized for 24 h (FreeZone^®^ 2.5, Labconco Corporation, Kansas City, MO, USA), powdered (Mini-Mill Cutting Mill, Thomas Scientific, Swedesboro, NJ, USA) and stored at −20 °C.

To prepare the hydrogel, powdered ECM samples were solubilized with 1.0 mg/ml pepsin in 0.01 N HCl (Sigma-Aldrich, Chemie GmbH Steinheim, GE) at a concentration of 10 mg ECM/ml and stirred at room temperature for 48 h to form a pre-gel solution (pH ~ 1.5–2.5). The pepsin-HCl ECM solution was neutralized to pH 7.4 with 0.1 N NaOH, isotonically balanced with 10x PBS, and diluted with 1x PBS to the final concentration of 8 mg/ml, which allows *in vivo* gelation^9,38^. The neutralized pre-gel was kept at 37 °C for ~120 min together with either genipin (Sigma) of required concentration solubilized in 50% DMSO to form genipin-crosslinked hydrogel (ECM/G) or with EDC (Sigma) of required concentration solubilized in H_2_O to form EDC-crosslinked hydrogel (ECM/D).

### Proteomic analysis

Proteins in ECM hydrogels and in the native umbilical cord tissue were identified by high-resolution LC–tandem mass spectrometry (MS/MS). The isolated proteins were digested with trypsin, concentrated and desalted using a trapping column (100 μm × 30 mm) filled with 3.5-μm X-Bridge BEH 130 C18 sorbent (Waters) and eluted onto an analytical column (Acclaim Pepmap100 C18, 3 µm particles, 75 μm × 500 mm; Thermo Fisher Scientific, Waltham, MA, USA). The analytical column outlet was directly connected to the Digital PicoView 550 ion source with PicoTip emitter SilicaTip (New Objective; FS360-20-15-N-20-C12). The analysis of the mass spectrometric RAW data files was carried out using the Proteome Discoverer software (Thermo Fisher Scientific; version 1.4) (Supplementary Materials).

### Crosslinking degree determination by TNBSA protocol

The crosslinking degree was determined by 2,4,6-trinitrobenzen sulfonic acid (TNBSA) protocol and was defined as the ratio of the consumed amino groups in the crosslinked samples to the free amino groups in the corresponding uncrosslinked samples^31^.

The amount of free amino groups in the test sample was determined by the optical absorbance of the solution at 335 nm recorded with a spectrophotometer (Infinite® 200 Pro, Tecan, Austria) using glycine at different concentrations (1.0, 2.0, 3.0, 4.0, and 5.0 mg/mL) as a standard. The samples were mixed in 0.01% TNBSA (Sigma) and 0.1 sodium bicarbonate (Sigma). After 2-hours incubation at 37 °C and adding 10% sodium dodecyl (Sigma) and 1 N HCl the amount of free amino groups in the tested samples was proportional to the optical absorbance of the solution. Both crosslinkers were tested also without gels to recognize no significant contribution of free crosslinkers to absorbance results.

The measured concentration (number of free amine moles in the sample per unit volume of dissolution) was divided by the sample number of moles and multiplied by the volume of dissolution, to obtain the free amine moles fraction in the sample. The degree of crosslinking was calculated following the equation (1):

1 $$ \% \,{\rm{crosslinking}}=\frac{{X}_{N{H}_{2}}-{X}_{N{H}_{2}cross}}{{X}_{N{H}_{2}}}\times 100$$

where ${X}_{N{H}_{2}}$is the mole fraction of free amines in uncrosslinked samples and ${X}_{N{H}_{2}}\,cross$ is the mole fraction of free amines remaining in 120 min crosslinked samples. Three replicates of each concentration were evaluated.

### Rheological measurement

Dynamic oscillatory shear tests were used to investigate the viscoelastic properties. ECM hydrogels crosslinked by 1 mM and 10 mM genipin or by 1 mM and 10 mM EDC were subjected to a sinusoidal deformation in a 40 mm parallel plate rheometer (Ares-G2, TA Instruments, New Castle, DE, USA) at 1 Pa stress and 10 °C to determine their mechanical response (displacement or strain) as a function of time. To prevent drying of the sample a thin layer of silicone oil was applied on the side of the sample which was not in contact with the plates, just before the measurement. The samples were placed between two plates of 25 mm or 12.5 mm in diameter. The bottom plate was a bowl with raised edge and defined space within the given diameter. The surface of both types of boards was finely roughened to prevent slipping. A frequency sweep test was run with the parameters of 5% strain, frequency 1–20 Hz, axial force: 0.01 N and temperature at 37 °C, 4 min duration. The test was repeated three times, at 12.5 mm and 25 mm diameter plates, with three independent samples in triplicate.

### Turbidity gelation measurement

The turbidimetric gelation kinetics were determined as previously described^39^ on a spectrophotometer (Infinite® 200 Pro, Tecan), which was pre-heated to 37 °C. ECM, ECM/G and ECM/D crosslinked by 1 mM and 10 mM genipin or EDC hydrogels were kept on ice at 4 °C until 100 µl were pipetted into each well of a 96 well plate, and inserted into a spectrophotometer. The absorbance was measured at 405 nm every 2 min for 120 min. Normalized absorbance, time to reach 50% and 95% maximal absorbance were determined as t_1/2_ and t_95_. The lag time (t_lag_) was defined as the point where a line representing the slope at log t_1/2_ intersects the turbidimetry baseline with 0% absorbance. The gelation rate (S) was defined as the slope of the linear region of the gelation curve. The measurements were repeated three times with three independent samples in triplicate (Table 1).

2 $$Normalized\,Absorbance=\frac{A-{A}_{0}}{{A}_{max}-{A}_{0}}$$

### 1-D degradation assay

ECM hydrogels (0.5 mL, each sample n = 3) were crosslinked with genipin (0.5; 1 and 5 mM) or EDC (1, 5 and 10 mM) in a 2 mL cryotube (Scientific Specialties, Inc., US) and allowed to form a gel for 1, 3, 24, 48 and 72 h at 37 °C. 1 mL of collagenase I (0.1 wt %, 163 U/mg, Sigma) in PBS with 0.9 mM CaCl_2_, was added on top of the gel and placed on a shaker at 150 rpm at 37 °C. After 3 h, the degradation medium was removed, the gels were rinsed with deionized water, frozen, lyophilized overnight, and weighed. The removal time was set by the degradation time for uncrosslinked ECM gels. The dry weights of the ECM hydrogels exposed to the collagenase were related to the weights of the control gel exposed to PBS for the same time; data are reported as % of the remaining weight.

### 3-D degradation assay

ECM hydrogels (0.5 mL, each sample n = 3) were crosslinked with genipin (0.5, 1 and 5 mM) or EDC (1, 5 and 10 mM) in a 2 mL cryotube and allowed to form a gel for 1 h or 48 h at 37 °C. The gels were then transferred into 24-well plates in PBS and exposed to collagenase I (0.1%, 1 mL, 163 U/mg) in PBS with 0.9 mM CaCl_2_ on a shaker (150 rpm); the control gels were exposed to PBS. After 30, 60, 90, or 120 min, the collagenase was removed, and the gels were washed with PBS on a shaker for 20 min. The remaining gel samples were collected, lyophilized overnight, and weighed. The dry weights of the ECM hydrogels exposed to collagenase, were related to the weight of the control ECM hydrogels in PBS; data are reported as % of remaining weight. Half-life times were calculated as the time required to degradation of 50% of gel original mass (Table 2).

### Mesenchymal stem cell culture

Human umbilical cord derived mesenchymal stem cells (MSCs) were used as described previously^40^ (Supplementary materials). To reveal the cytotoxicity of the crosslinkers, the cells were seeded into a 96-well plate (5000 cells/cm^2^ in 100 µl media) with an increasing concentration of genipin or EDC 0.5–10 mM in the media. After 4 h, 1 day, 4 days and 7 days of the culture, 10 µl of Alamar Blue reagent was added to each well containing 100 µl culture media and incubated for 3 hours at 37 °C. The fluorescence was measured using a Tecan-Spectra plate reader at 580 nm (emission) and 540 nm (excitation), the signal from the hydrogels without the cells was used for the fluorescence subtraction.

To determine the biocompatibility of the crosslinked hydrogels, the hydrogels were pipetted into a 96-well plate (90 µl/well) and seeded with cells (5000 cells/cm^2^ in 100 µl media). The cell proliferation was measured using Alamar Blue assay after 4 h, 3 days, 7 days and 14 days of the culture. Each type of hydrogel was seeded in triplicate. Six independent experiments of three hydrogel batches were performed for each concentration. After 14 days in culture, the cells were visualized by Live/Dead Staining (Sigma, Supplementary Fig. 1).

### ECM hydrogel contraction in 3D culture

In 3D cultures, MSC suspension in PBS was mixed with a neutralized liquid pre-gel solution for a final cell concentration of 2.5 × 10^6^ cells/ml and crosslinked by 0.5–5 mM genipin and 1–10 mM EDC. 200 µl of the suspension was transferred inside the cylindrical mould (0.8 cm, Scaffdex, Tampere, FI) placed in the 24-well plates, and incubated at 37 °C for 45 min, to form a mechanically stable hydrogel disc seeded with the cells. Following this, the seeding moulds were removed, 1 ml of culture media was added to support living cells in the gel, and the hydrogel discs were incubated at 37 °C, and imaged after 0, 4, 24, and 48 h of the culture to quantify gel contraction. ImageJ (NIH) tracing module was used to measure the hydrogel areas at each time point and the values are shown as a percentage of the initial hydrogel area. The hydrogels were seeded in triplicate, and three hydrogel batches were used for the analysis. As a control, ECM hydrogel discs without cells were maintained in the culture under the same conditions.

To determine 3-D cell viability, ECM and ECM crosslinked by 0.5–5 mM genipin and 1–10 mM EDC were mixed with MSCs (5 × 10^4^ in 20 µl of the hydrogel) and pipetted into a 96-well plate. After 45 min of gelation, the medium (100 μm) was added, and the cell proliferation was measured using Alamar Blue assay after 4 h, 1 and 2 days of the culture. The data were normalized to the initial viability values obtained after 4 h for the 3-D MSC culture in the uncrosslinked ECM.

To evaluate cell infiltration, 50 µl of ECM, ECM/G and ECM/D crosslinked by 1 mM genipin or EDC, were pipetted into the 96-well plate and let mature for 24 h. MSCs (5 × 10^3^) were seeded on the surface of the hydrogels and cultured for 7 days. Cells were fixed with 4% paraformaldehyde in PBS and stained with immunofluorescent labelling for Alexa-Fluor 568 Phalloidin (1:400; Molecular Probes, Eugene, OR, USA). 3-D images were taken using the confocal microscope Zeiss LSM 5 DUO (Carl Zeiss, Jena, GE).

### Axonal growth on the ECM hydrogels

Dorsal root ganglia (DRGs) were dissected from adult (2 months) male Wistar rats (Velaz, Unetice, CZ). The neurons were dissociated with 0.2% collagenase from Clostridium histolyticum and 0.1% trypsin (both from Sigma) and then centrifuged through 15% bovine serum albumin (BSA, Sigma). DRGs were cultured on coverslips coated with ECM, ECM/G, ECM/D hydrogels (300 µl, EDC/genipin concentration 1 mM) in DMEM (ThermoFisher, Waltham, MA, USA) supplemented with penicillin–streptomycin–fungizone (1%, Lonza, Basel, CH), ITS + (1%), NGF (10 ng/ml) and mitomycin C (0.5 µg/ml, all from Sigma) for 2 days. Cells were fixed with 4% paraformaldehyde in PBS and stained with anti-beta-III-Tubulin (1:1200; Abcam, Cambridge, UK) and goat anti-mouse IgG Alexa Fluor® 488 (1:400, Life Technologies, Eugene, OR, USA), and imaged on a fluorescent microscope AxioCam HRc Axioskop 2 Plus (Zeiss, Jena, GE) a 20x objective and ImageJ software. For the analysis, a percentage of neurons with neurites longer than the cell body were calculated from 3 wells and 3 independent areas in each well. The experiment was repeated three times.

### Neural stem cells growth and differentiation

The human fetal neural stem cell (NSC) line SPC-01, which is a conditionally immortalized cell line, was generated from 8-week-old human fetal spinal cord as described previously^41,42^. SPC-01 cells were cultured in tissue-culture flasks freshly coated with laminin 10 µg/ml (Sigma) in DMEM/F12 (GIBCO, Life Technologies, Grand Island, NY, USA) for 2 hours at 37 °C. Growth media comprising DMEM/F12 supplemented with human serum albumin (0.03%, Baxter Healthcare Ltd., Norfolk, UK), human apo-transferrin (100 μg/ml), putrescine DiHCl (16.2 μg/ml), human recombinant insulin (5 μg/ml), progesterone (60 ng/ml), L-glutamine (2 mM), sodium selenite (40 ng/ml), 4-OHT (100 nM) all from Sigma, human EGF (20 ng/ml), human bFGF (10 ng/ml) (PeproTech, London, UK) and primocin 100 µg/ml (InvivoGen, San Diego, CA, USA), was changed 3 times per week.

A suspension of 30 000 SPC-01 cells was seeded on laminin coated glass coverslips or 200 000 cells on ECM gels and cultured in a 24-well plate with a cultivation medium for 7 and 21 days. The cells were then fixed in 4% paraformaldehyde in PBS for 15 min and washed with PBS. The growth and differentiation of SPC-01 cells were analysed using immunofluorescent labelling for Alexa-Fluor 568 Phalloidin (1:400; Molecular Probes, Eugene, OR, USA), glial fibrillary acidic protein (GFAP, 1:800; mouse monoclonal IgG1 conjugated with Cy3; Sigma), beta-III-Tubulin (1:1200; rabbit monoclonal IgG; Abcam), NG2 chondroitin sulphate proteoglycan (1:400; rabbit polyclonal IgG; Abcam). Goat anti-rabbit IgG (H + L) conjugated with AlexaFluor 594 (1:400; Life Technologies) was used for visualization of beta-III-Tubulin and NG2 antibodies. The nuclei were visualized using 4′,6′, -diamidino-2-phenylindol (DAPI) fluorescent dye (1:1000; Life Technologies). Images were taken using the confocal microscope Zeiss LSM 5 DUO. The cell growth was determined from 10 random fields of view in each gel and analysed using ImageJ.

### Injection of genipin crosslinked hydrogels into a rat model of cortical photothrombotic lesion

To test biocompatibility of ECM/G hydrogel *in vivo*, a focal brain photochemical lesion was created in the motor cortex in rats. Male Wistar rats (330 ± 30 g) (Velaz) were maintained at 22 °C on a 12 h light/dark schedule and given water and food *ad libidum*. To create the lesion, the animal was placed into the stereotactic apparatus under isoflurane (3%) anaesthesia, the scalp incision was created in the midline and the pericranial tissue was dissected to expose the bregma. Focal cerebral ischaemia was performed according to^43^ as follows: Bengal Rose (Sigma) was injected via the right femoral vein (0.08 g/ml saline; 1 µl/g), and the skull was illuminated above the primary motor cortex (2 mm rostral and 2 mm dextrolateral to the bregma) with a fiber optic bundle of a cold light source (KL 1500 LCD; Zeiss) for 10 minutes. The skin overlying the cranium was then sutured.

Seven days after the focal cerebral ischaemia, a small opening in the lesion site was drilled into the scull of the animal and 10 µl of ECM (n = 5) or ECM/G (n = 5) in the form of pre-gel was injected into the lesion (in a depth of 2 mm) using a Hamilton syringe (Hamilton Company, Bonaduz, Switzerland) and stereotactic apparatus.

The animals were sacrificed 1 day and 14 days after the implantation with an overdose of anaesthesia and perfused with 4% paraformaldehyde in 0.1 M PBS intracardially. The brains were removed, fixed in 4% paraformaldehyde for 10 days and cut in frozen mode (local temperature −24 °C). Coronal slides, 40 µm thick, were stained for cell nuclei with DAPI (1:1000; Life Technologies), rabbit monoclonal IgG to vimentin (1:200, Abcam), mouse monoclonal IgG1 to CD68 (ED1; 1:150, Abcam), goat polyclonal IgG to CD206 (c-20; 1:250, Santa Cruz, Heidelberg, Germany) or mouse monoclonal IgG1 to collagen I (1:1000, COL-I, Abcam) diluted in 0.1 M PBS containing goat (or donkey – depending on the host organism of secondary antibodies) serum (1:10 both; Sigma) and Triton X-100 (0.1%) overnight in 4 °C. A staining solution lacking Triton-X was used only in the case of extracellular anti-collagen staining. As secondary antibodies, goat anti-mouse IgG conjugated with AlexaFluor 594 (1:400) for collagen I, donkey anti-mouse IgG conj. with AlexaFluor 488 (1:400) for CD 68 and donkey anti-goat IgG conj. with AlexaFluor 594 (1:400;) for CD206 (all from Life Technologies) were used.

Fluorescent images were taken using confocal microscope Zeiss LSM 5 DUO. The relative number of microglia/ macrophages (CD68+) and (CD206+) in the area surrounding the lesion was determined from sections taken by fluorescent microscope (Leica, Olympus Optical, Hamburg, Germany) using a 20x objective and ImageJ software. The quantity of the ED1 positive and CD206 positive macrophages was related to the number of all cell nuclei stained for DAPI in the area surrounding the lesion.

All experiments on the animals were performed in accordance with the European Communities Council Directive of 24^th^ November 1986 (86/609/EEC), regarding the use of animals in research and were approved by the Ethical Committee of the Institute of Experimental Medicine Academy of Sciences Czech Republic, Prague.

### Statistical analysis

Data were presented as mean ± standard error mean (SEM). The statistical significance was analysed using one-way ANOVA with Tukey’s multiple comparison post hoc analysis (GraphPad Prism, San Diego, CA, USA) with a level of p < 0.05 considered statistically significant.

## Supplementary information

Supplementary materials
