# Supplementary material for: Genipin and EDC crosslinking of extracellular matrix hydrogel derived from human umbilical cord for neural tissue repair
Source: Sci Rep. 2019 Jul 23;9:10674. doi: 10.1038/s41598-019-47059-x (PMC6650505; doi:10.1038/s41598-019-47059-x)
Supplement: Supplementary file 1 — Supplementary materials [file 41598_2019_47059_MOESM1_ESM.pdf]

Supplementary materials

Genipin and EDC crosslinking of extracellular matrix hydrogel derived from human umbilical cord for neural tissue repair

Karel Výborný[1, 2], Jana Vallová[1, 2], Zuzana Kočí[1], Kristýna Kekulová[1, 2], Klára Jiráková[1], Pavla Jendelová[1], Jiří Hodan[3], Šárka Kubinová[1]

[1]*Institute of Experimental Medicine of the Czech Academy of Sciences, Prague, Czech Republic*

[2]*2nd Medical Faculty, Charles University, Prague, Czech Republic*

[3]*Institute of Macromolecular Chemistry of the Czech Academy of Sciences, Prague, Czech Republic*

Corresponding author:

Sarka Kubinova, PhD

Institute of Experimental Medicine CAS, Videnska 1083, 1420 Prague, Czech Republic

E-mail: sarka.kubinova@iem.cas.cz

Phone: +420 241 062 635

Proteomic analysis

Sample preparation for liquid chromatography mass spectrometry (LC-MS/MS) analyses

The native tissue samples (n = 2) and ECM hydrogel samples (n = 3) were used for the analysis. Tissue samples were homogenized using cryomill (6870, SPEX SamplePrep) by the manufacturer recommended procedure. Proteins from powdered tissue sample were extracted using SDT buffer (4 % SDS, 0.1M DTT, 0.1M Tris/HCl pH 7.6) in the presence of acid washed glass beads (G4649, Sigma) for 30min at 95°C while shaking (thermomixer comfort, Eppendorf). Protein solution was cleared by centrifugation (20,000×g; Eppendorf). Protein extraction from decellularized tissue samples was done using the same procedure without powder preparation step.

All protein solutions were processed by filter-aided sample preparation (FASP) method [1]. The samples were mixed with 8M UA buffer (8M urea in 100 mM Tris-HCl, pH 8.5), loaded onto the Microcon device with MWCO 10 kDa (Merck Millipore) and centrifuged at 7,000× g (the next centrifugation steps done at 14,000× g) for 30 min at 20°C. The retained proteins were washed with 200 μL UA buffer. The final protein concentrates kept in the Microcon device

were mixed with 100 μL of UA buffer containing 50 mM iodoacetamide and incubated in the dark for 20 min. After the next centrifugation step, the samples were washed three times with 100 μL UA buffer and three times with 100 μL of 50 mM NaHCO3. Trypsin (sequencing grade, Promega) was added onto the filter and the mixture was incubated for 18 h at 37°C. The tryptic peptides were finally eluted by centrifugation followed by two additional elutions with 50 μL of 50 mM $NaHCO_3$. Resulting peptides were extracted into LC-MS vials by 2.5% formic acid (FA) in 50% acetonitrile (ACN) and 100% ACN with addition of polyethylene glycol (final concentration 0.001%) [2] and concentrated in a SpeedVac concentrator (Thermo Fisher Scientific).

LC-MS/MS analysis of peptides

LC-MS/MS analyses of peptide mixtures were done using RSLCnano system connected to Orbitrap Elite hybrid spectrometer (Thermo Fisher Scientific). Prior to LC separation, tryptic digests were online concentrated and desalted using trapping column (100 μm × 30 mm) filled with 3.5-μm X-Bridge BEH 130 C18 sorbent (Waters). After washing of trapping column with 0.1% FA, the peptides were eluted (flow 300 nl/min) from the trapping column onto an analytical column (Acclaim Pepmap100 C18, 3 μm particles, 75 μm × 500 mm; Thermo Fisher Scientific) by 100 min nonlinear gradient program (1-56% of mobile phase B; mobile phase A: 0.1% FA in water; mobile phase B: 0.1% FA in 80% ACN). Equilibration of the trapping column and the column was done prior to sample injection to sample loop. The analytical column outlet was directly connected to the Digital PicoView 550 (New Objective) ion source with PicoTip emitter SilicaTip (New Objective; FS360-20-15-N-20-C12). ABIRD (Active Background Ion Reduction Device) was installed.

MS data were acquired in a data-dependent strategy selecting up to top 10 precursors based on precursor abundance in the survey scan (350-2000 m/z). The resolution of the survey scan was 60 000 (400 m/z) with a target value of 1×10^6 ions, one microscan and maximum injection time of 200 ms. HCD MS/MS spectra were acquired with a target value of 50 000 and resolution of 15 000 (400 m/z). The maximum injection time for MS/MS was 500 ms. Dynamic exclusion was enabled for 45 s after one MS/MS spectra acquisition and early expiration was disabled. The isolation window for MS/MS fragmentation was set to 2 m/z.

The analysis of the mass spectrometric RAW data files was carried out using the Proteome Discoverer software (Thermo Fisher Scientific; version 1.4) with in-house Mascot (Matrixscience, London, UK; version 2.6.1) and Sequest search engines utilization. MS/MS ion searches were done at first against modified cRAP database (based on

http://www.thegpm.org/crap/; 111 sequences in total) containing protein contaminants like keratin, trypsin etc. MS/MS spectra assigned by Mascot search engine to any cRAP protein peptide with Mascot ion score >30 were excluded from the next database searches. Final database searches were done against UniProtKB proteome database for Homo sapiens (taxonomy ID 9606; database version 2017-07, number of proteins 20,975). Mass tolerance for peptides and MS/MS fragments were 10 ppm and 0.05 Da, respectively. Oxidation of methionine, deamidation (N, Q) and acetylation (protein N-terminus) as optional modification, carbamidomethylation (C) as fixed modification and one enzyme miss cleavage were set for all searches. Percolator was used for post-processing of the search results. Peptides with q-value <0.01, rank 1 and with at least 6 amino acids were considered only. Proteins matching the same set of peptides were reported as protein groups. Protein groups/proteins were reported only if they had at least one unique peptide. Label-free quantification using protein area calculation in Proteome Discoverer was used ("top 3 protein quantification" [3]). Protein group reports from all individual samples were combined into a single supergroup (SG) report where each SG is list of proteins reported within a single protein group in at least single sample report.

Supplementary Table 1: The most represented proteins in the ECM and native umbilical cord tissue

Core ECM proteins	ECM (ppm*)	Native WJ (ppm*)	% (ECM/ native tissue)
Collagen alpha-1(I) chain	396521	101666	390
Collagen alpha-2(I) chain	260669	121210	215
Collagen alpha-1(III) chain	195378	39022	501
Collagen alpha-1(II) chain	80283	17415	461
Collagen alpha-1(V) chain	14552	18230	80
Collagen alpha-2(V) chain	7349	9535	77
Collagen alpha-2(XI) chain	6655	6954	96
Collagen alpha-1(XI) chain	4807	12002	40
Collagen alpha-2(IV) chain	4172	5663	74
Collagen alpha-1(IV) chain	3562	6839	52
Collagen alpha-3(VI) chain	2842	16742	17
Collagen alpha-2(VI) chain	2338	9260	25

Collagen alpha-1(VI) chain	1558	12143	13
Collagen alpha-1(XII) chain	1088	4358	25
Fibrillin-1	2542	6265	41
Fibronectin	1793	7808	23
Fibrillin-2	1218	3305	37
Tenascin	397	2575	15
Laminin subunit beta-1	98	398	25
Laminin subunit gamma-1	90	702	13

**Cytoskeleton/cytosolic/ membrane proteins**	**ECM (ppm*)**	**Native WJ (ppm*)**	**% in ECM vs. native tissue**
Actin. aortic smooth muscle	2028	57420	4
Actin. cytoplasmic 2	1992	36727	5
Tropomyosin beta chain	1061	6720	16
Tropomyosin alpha-1 chain	1059	6718	16
Myosin-11	892	6095	15
Caveolin-1	310	1532	20
Myosin-9	263	4303	6
Myosin-10	209	3706	6
Filamin-A	144	9728	1
Glyceraldehyde-3-phosphate dehydrogenase	27	7368	0

*ppm values from adjusted SG Areas

Supplementary Table 2: Turbidimetric measurement summary

	ECM	ECM/G 1 mM	ECM/G 10 mM	ECM/D 1 mM	ECM/D 10 mM
**t_{lag} (sec)**	148.95 ± 52.11	142.25 ± 35.01	132.25 ± 47.45	250.26 ± 35.78	248.20 ± 38.11
**$t_{1/2}$ (min)**	11.30 ± 1.29	34.94 ± 1.03	36.01 ± 1.39	30.03 ± 3.22	46.32 ± 5.25
**t_{95} (min)**	48.03 ± 8.92	97.74 ± 2.48	98.25 ± 1.98	96.25 ± 4.25	99.25 ± 6.25
**delta S (min^{-1})**	0.10 ± 0.01	0.04 ± 0.01	0.05 ± 0.05	0.37 ± 0.05	0.35 ± 0.04

Mesenchymal stem cell culture

Human umbilical cord derived mesenchymal stem cells (MSCs) were used to evaluate the biocompatibility of the material. Fresh human umbilical cord samples were collected from healthy full-term neonates after spontaneous delivery with the informed consent of the donors using the guidelines approved by the Institutional Ethics Committee at University Hospitals in Pilsen and Prague, Czech Republic. About 10-15 cm per umbilical cord were aseptically transported into sterile PBS (IKEM, Prague, Czech Republic) with an antibiotic-antimycotic solution (Sigma) at 4°C. After the removal of blood vessels, the remaining tissue was chopped into small pieces (1-2 mm^3) and transferred to 10 cm Nunc culture dishes (Schoeller, Prague, CZ) containing the complete medium alpha-MEM (EastPort, Prague, CZ) supplemented with 5% platelet lysate (IKEM) and gentamicine 10 µg/ml (Sandoz, Prague, CZ), and cultivated at 37°C and 5% CO_2 in a humidified atmosphere. On day 10, the explants were removed from the culture dishes and the remaining adherent cells were cultured for 3 weeks or until 90% confluence. The medium was changed twice a week. The cells of the 3rd passage were identified by flow cytometry (FACSAria™, Becton Dickinson, San Jose, California) to confirm their purity. The following antibodies against human antigens were used: CD34, CD45, CD105, (Exbio, Vestec, CZ); CD29, CD73, CD90, CD271, CD31, HLA-ABC and CD235a (BD Pharmingen, San Jose, CA, USA); CD133 (Miltenyi Biotec, Bergisch Gladbach, GE). Data analysis was performed using BD FASCDiVa software. The cells in the 6th passage were analyzed for ECM hydrogel evaluation [4].

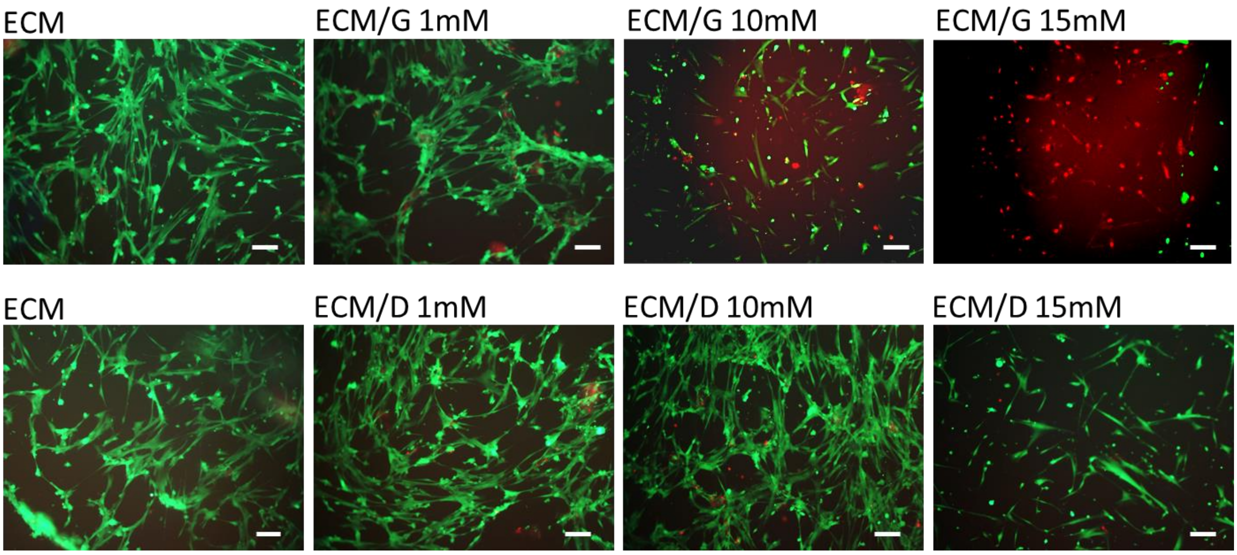

Supplementary Figure 1. Cell growth on ECM hydrogels after 2 weeks of the culture. The cell are stained by live/dead assay. Green channel depicts live cells and red channel depicts compromised/dead cells. Scale bar = 100 µm.

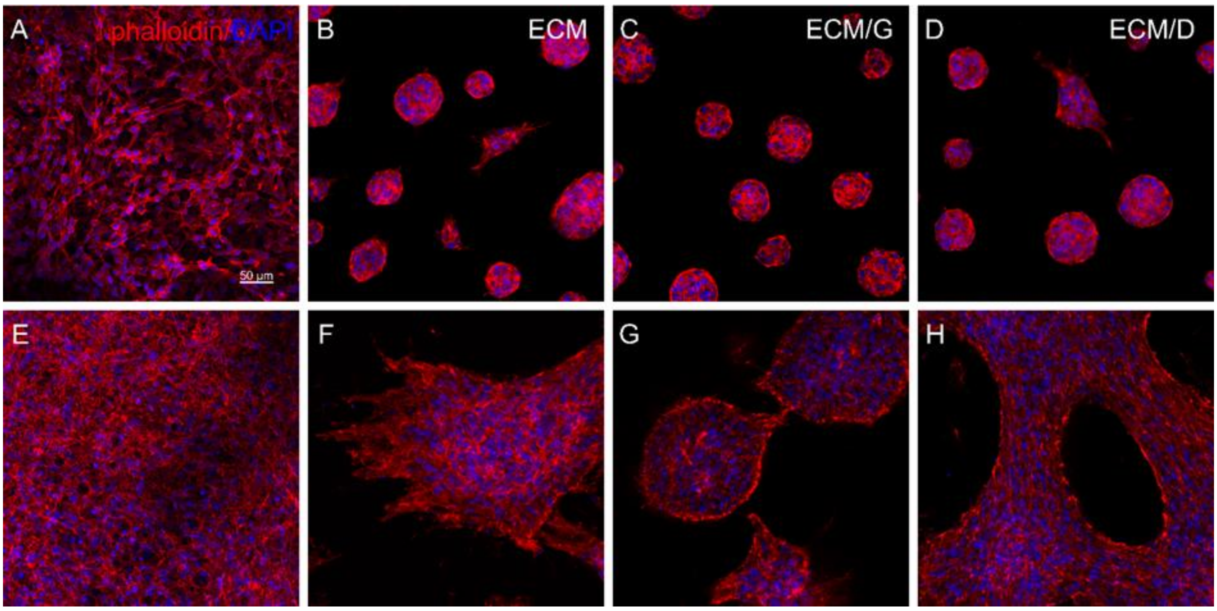

Supplementary Figure 2. Neural stem cells cultured (A, E) on laminin coated glass coverslips, (B, F) uncrossliked ECM, (C, G) ECM/G and (D, H) ECM/D after (A-D) 1 week and (E-H) 3 weeks. Cells were stained with phalloidin (red) and DAPI (blue). Scale bar: 50 µm.

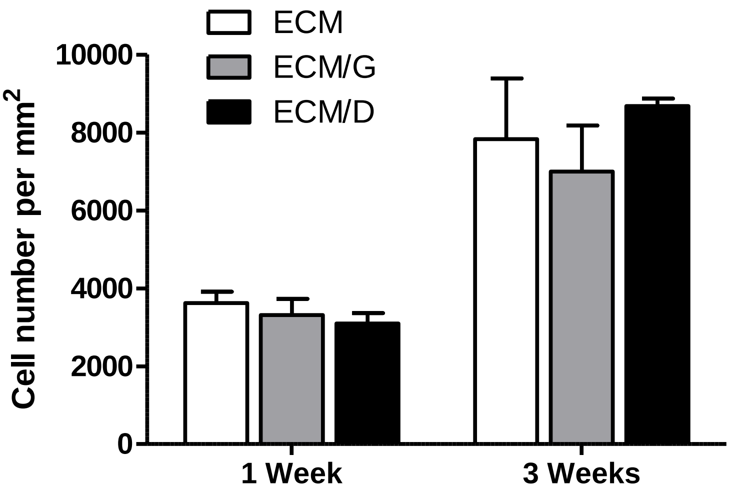

Supplementary Figure 3. The density of SPC-01 cells seeded on ECM gels after 1 week and 3 weeks. There was no statistically significant difference between groups. (n = 10)

References:

1 Wisniewski, J. R., Ostasiewicz, P. & Mann, M. High recovery FASP applied to the proteomic analysis of microdissected formalin fixed paraffin embedded cancer tissues retrieves known colon cancer markers. *J Proteome Res* **10**, 3040-3049, doi:10.1021/pr200019m (2011).

2 Stejskal, K., Potesil, D. & Zdrahal, Z. Suppression of peptide sample losses in autosampler vials. *J Proteome Res* **12**, 3057-3062, doi:10.1021/pr400183v (2013).

3 Silva, J. C., Gorenstein, M. V., Li, G. Z., Vissers, J. P. & Geromanos, S. J. Absolute quantification of proteins by LCMSE: a virtue of parallel MS acquisition. *Mol Cell Proteomics* **5**, 144-156, doi:10.1074/mcp.M500230-MCP200 (2006).

4 Krupa, P. *et al.* The Effect of Human Mesenchymal Stem Cells Derived from Wharton's Jelly in Spinal Cord Injury Treatment Is Dose-Dependent and Can Be Facilitated by Repeated Application. *Int J Mol Sci* **19**, doi:10.3390/ijms19051503 (2018).
